# Research progress and application of single-cell sequencing in head and neck malignant tumors

**DOI:** 10.1038/s41417-023-00691-2

**Published:** 2023-11-15

**Authors:** Siyuan Qu, Mengdan Gong, Yongqin Deng, Yizhen Xiang, Dong Ye

**Affiliations:** Department of Otorhinolaryngology-Head and Neck Surgery, The Affiliated Lihuili Hospital, Ningbo University, Ningbo, 315040 Zhejiang China

**Keywords:** Cancer genomics, Tumour heterogeneity

## Abstract

Single-cell sequencing (SCS) is a technology that separates thousands of cells from the organism and accurately analyzes the genetic material expressed in each cell using high-throughput sequencing technology. Unlike the traditional bulk sequencing approach, which can only provide the average value of a cell population and cannot obtain specific single-cell data, single-cell sequencing can identify the gene sequence and expression changes of a single cell, and reflects the differences between genetic material and protein between cells, and ultimately the role played by the tumor microenvironment. single-cell sequencing can further explore the pathogenesis of head and neck malignancies from the single-cell biological level and provides a theoretical basis for the clinical diagnosis and treatment of head and neck malignancies. This article will systematically introduce the latest progress and application of single-cell sequencing in malignant head and neck tumors.

## Background

Head and neck tumors account for approximately 5% of malignant tumors, which poses a serious threat to human health. To date, most of our understanding of driver mutations and abnormal regulation of head and neck malignancies has derived from data from sequencing technology.

Since its first appearance, single-cell sequencing (SCS) technology has developed rapidly, making it possible to analyze the genome of a single cell. In the field of tumor research, SCS technology reveals the heterogeneity within tumors, differentiation and evolution of cancer cells, cancer invasion and metastasis, treatment resistance, and tumor microenvironment by studying single nucleotide polymorphism (SNP), copy number variation, genomic structure variation at the single-cell DNA level, gene expression variation at the RNA level, gene fusion, alternative splicing, DNA methylation, and histone modification. Capturing the diversity of tumor-specific cells and cell subsets has promoted the development of basic research and clinical application in cancer [1, 2].

This article will summarize the latest progress of SCS in malignant tumors of the head and neck and will also explore the application and development prospects of SCS technology in cancer research and clinical practice.

## Single-cell sequencing technology

**Fig. 1:**
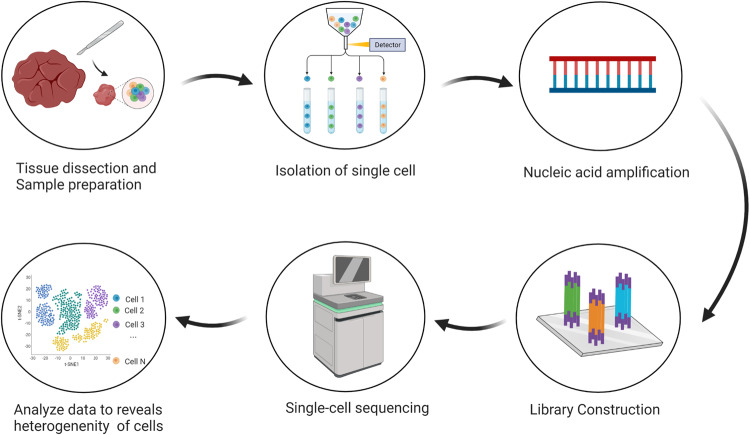
Single-cell sequencing flow chart. The main process of single-cell sequencing technology includes sample preparation, single-cell isolation, nucleic acid amplification, library construction, single-cell sequencing and data analysis.

SCS sequencing technology was first reported by Tang et al. in 2009. It has been widely considered as the technology with the most potential. After ten years of rapid development, single-cell sequencing technology has evolved from initially isolating single cells and constructing independent libraries to detect a small number of cells. Now, the emergence of single-cell recognition and new cell separation technologies based on barcode tags has brought new discoveries to tumor biology, immune oncology, and clinical treatment [3]. The main processes involved in SCS will be described in detail below (Fig. 1).

### Acquisition of single cells

Acquisition of single cells is the first and key step in the SCS technology. Separation of single cells involves the separation of single cells directly from the material to be separated. It requires not only accurate and effective acquisition of single cells but also a certain proportion and number of active cells.

#### Micromanipulation

Micromanipulation is a method of manually extracting individual cells from suspension based on cell morphology and fluorescence color characteristics using a microscope-guided capillary pipette. However, this technology can process only specific cells in one sample at a time, so it only suitable for processing samples with a small number of cells [4].

#### Laser capture microdissection

Laser capture microdissection (LCM) is a relatively simple and inexpensive method with high repeatability and sensitivity. LCM directly lyses fixed tissue cells under the microscope with ready-made reagents without RNA purification. LCM also has the advantage that the tissue sections remain intact, thus preserving the location information of each cell, and the accuracy is extremely high, even allowing the separation of subcellular components. The disadvantages of LCM are low flux and high cost [5].

#### Fluorescence-activated cell sorting (FACS)

Fluorescence-activated cell sorting (FACS) is based on the characteristics of fluorescent labeling of specific cells by flow cytometry and is recognized and recovered according to cell DNA content (fluorescence) and scattering signals. Because of its high speed and high throughput, it has become a common method for single-cell separation. The main limitation of FACS is that a large number of sample cells are required as starting materials [6].

#### Microfluidic technology

Microfluidic technology is a technology that uses the principle of fluid mechanics to separate and capture single cells in micron-diameter channels. It can use a small number of samples and chemical reagents to identify and separate single cells. It can detect rare cell types and makes parallel sequencing of mRNA in large-scale single cells possible [7, 8]. Currently, Fluidigm C1 and 10x Genomics are the most commonly used microfluidic commercial platforms.

### Single-cell genome sequencing

Single-cell DNA sequencing (scDNA-seq) is used to amplify the DNA of isolated single cells and perform high-throughput sequencing to reveal the differences between cell populations and the relationship between cell evolution [9].

#### PCR amplification

PCR is a whole genome amplification method using degenerate oligonucleotide primers. The invention of PCR greatly reduces the number of target biomaterial required for genetic research. This method is simple and inexpensive, but the lack of genomic DNA template sometimes leads to a decrease in the reliability of the results of downstream applications. Therefore, the quality of starting DNA is critical to the results [10, 11].

#### Multiple displacement amplification

Multiple displacement amplification (MDA) is the most commonly used whole genome amplification (WGA) method. MDA uses polymerase with strong chain displacement activity (such as phi29 DNA polymerase) and exonuclease to exponentially amplify single-stranded DNA into hyperbranched structure, so as to produce more uniformly amplified high molecular weight products on the template, provide higher genome coverage and lower false positive rates. However, the continuous amplification of polymerase tends to produce bias and reduce the uniformity of amplification [11, 12].

#### Multiple annealing and looping-based amplification cycles

Multiple annealing and looping-based amplification cycles (MALBAC) combines MDA with PCR technology. MALBAC replicates the products of the initial strand replacement synthesis step by quasi-linear amplification and hybridizes into a loop at its 3 and 5 ends through complementary sequences. This method reduces the experimental deviation related to nonlinear amplification and shows higher efficiency and sensitivity in determining gene copy number variation (CNV) and single nucleotide variation (SNV), but also presents the problem of high false positive rate [13].

### Single-cell transcriptome sequencing

Single-cell transcriptome sequencing (scRNA-seq) uses high-throughput gene sequencing technology used to sequence and analyze all transcriptome information in cells. RNA transcription and stability are strictly regulated by physiological and pathological stimuli. Any change may be reflected at the RNA level, causing differences between cells [14].

#### Tang-seq

Tang-seq is based on poly (A) tail amplification. Free primers are removed by exonuclease, and the poly(A) tail is added to the 3’end of the first-strand cDNA by terminal deoxynucleotidyl transferase. Single-cell cDNA PCR cycle amplification is used to construct a sequencing library. The disadvantage is the premature termination of reverse transcription, which significantly reduces the transcriptional coverage at the 5’end [3].

#### Cell expression by linear amplification and sequencing (CEL-seq)/seq2

CEL-seq/seq2 is a linear amplification method of transcription in vitro. Barcode primers are used to reverse-transcribe the cells in a single tube. After the second strand was synthesized, all cDNAs are mixed for PCR amplification. This method can reduce amplification bias and has higher repeatability and sensitivity compared to PCR-based methods, but it has sequence preference in both linear amplification and PCR [3, 15].

#### Switching mechanism at the 5’end of the RNA transcript(Smart-seq)/seq2

Smart-seq/seq2 belongs to the template conversion method, which uses special primers to combine with Moloney murine leukemia virus (M-MLV) reverse transcriptase enzyme to add several C bases to the 3’end of the newly synthesized cDNA. The M-MLV enzyme guides the DNA polymerase using cDNA as a template to obtain full-length double-stranded cDNA. Although time-consuming and laborious, it reduces the 3’end coverage deviation caused by incomplete reverse transcription, while improving the coverage of cross transcripts [8, 15].

### Single-cell epigenetic sequencing

Single-cell epigenetic sequencing technology has mainly been used to explore the impact of genetic information beyond the genome and transcriptome on cell function. There are also great differences in the epigenetic status of homologous cell populations, so it is of great significance to develop high-throughput methods to detect epigenomes.

#### Single-cell DNA methylation sequencing

During normal cell development, DNA methylation exists in the DNA sequence as a chemical or protein tag, which can regulate cell growth and metabolism. Single-cell DNA methylation sequencing methods mainly include single-cell simplified bisulfite sequencing (scRRBS-seq) and single-cell bisulfite sequencing (scBS-seq). scRRBS-seq uses restriction endonuclease to cut the genome and enrich the promoter and CpG fragment for sequencing. scBS-seq performs PCR with specific primers after bisulfite treatment, so that the unmethylated cytosine in the product is replaced. Therefore, the absolute quantification of single cytosine resolution and genome-wide DNA methylation level can be provided [3, 16].

#### Single-cell histone modification sequencing

To explore the function of histone modification in different cells, four methods including single-cell chromatin immunoprecipitation sequencing, single-cell chromatin integrated labeling post-sequencing, targeted single-cell cleavage and labeling, and single-cell chromatin immunocleavage post-sequencing have been developed. Histone modification can change the interaction between DNA and protein and changes the configuration of chromatin. Through these methods, the cell heterogeneity and differentiation status of the tumor can be identified [3, 17].

#### Single-cell chromatin structure sequencing

By studying the open chromatin regions of cells in a specific state, we can understand their transcriptional regulation at the DNA level. Because cis regulatory elements are usually highly sensitive to active nuclease or transposase, high-throughput single-cell sequencing technology is based on highly active Tn5 transposase and the chromatin open region. Thus, single-cell assays for transposase accessible chromatin using sequencing (scATAC-seq) have been developed [18]. ASAP-seq, an extension of scATAC-seq, simultaneously detects a large number of proteins and mitochondrial DNA (mtDNA) in thousands of single cells, and is widely used in epigenomics research [19].

## Function of single-cell sequencing

### Revealing tumor heterogeneity

Intratumoral heterogeneity of tumor cells is caused by epigenetic and genetic changes of tumor stem cells [20]. SCS technology helps us understand the impact of heterogeneity on tumor behavior and clinical outcomes.

**Fig. 2:**
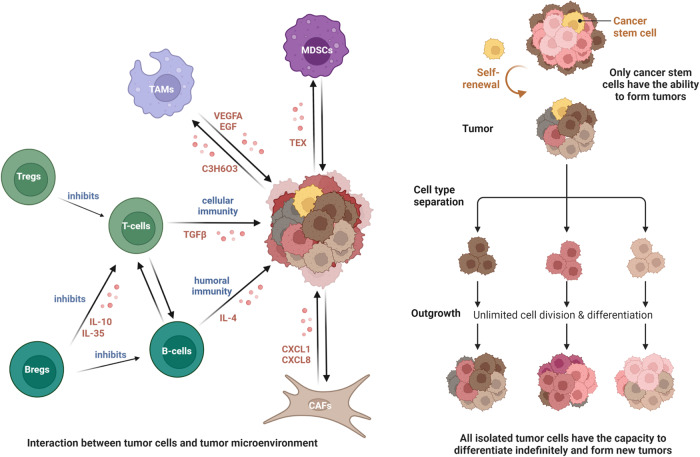
The origin of tumor and the role of tumor microenvironment. The interaction between malignant and nonmalignant cells generates tumor microenvironment, which affects the development and invasion of cancer. Tregs inhibit antitumor immunity by killing effector T cells; Bregs inhibit B cells and promote the proliferation of Tregs by releasing IL-2 and IL-35; TAMs promote tumor angiogenesis by producing VEGF-A and EGF, and the lactic acid produced by tumor cells in turn promotes the formation of TAMs; CAFs secrete CXCL1 and CXCL8 to promote the metastasis of tumor cells; MDSCs can be activated and expanded under the action of tumor-derived exosomes (TEXs) to help tumor angiogenesis and immune escape. Tumor stem cells have the ability of self-replication and multi-cell differentiation, which is the root of tumor formation and recurrence. In the process of cancer progression, driver mutations have a selective advantage, and different subclones of each gene can show significant phenotypic variation.

Most cancers evolve from a series of clonal expansions driven by an initial mutation in a single cell, which can result in each mutation being present in the same cancer cell clone. With tumor growth, additional drivers and gene mutations continue to appear. Obtaining additional mutations of driver tumor cells and initiating clonal expansion, which will generate a subpopulation of cells with mutations (Fig. 2). Due to the fact that these mutations are not shared by all cells in the tumor, using traditional population sequencing methods to develop clinical treatment plans may not yield good results in all patients. Massively parallel sequencing data analysis can infer the subclonal composition of tumors by identifying cell populations with shared mutations, determine the subclonal patterns of cancer characterized by driver gene mutations, fusion, structural variation, and CNAs, and the dynamic changes during subclonal amplification and mutation [21, 22].

Studying the gene map of cancer will help researchers better understand the molecular mechanism of disease occurrence and development and provide a theoretical basis for disease treatment and prediction.

### The tumor microenvironment

SCS technology has been used in the study of the tumor microenvironment (TME). Through the description of the single-cell transcriptome landscape, we can better understand the progression of the tumor. The TME refers to the cellular environment in which tumor or cancer stem cells exist. It can be divided into two parts: the immune microenvironment that contains immune cells and the non-immune microenvironment dominated by fibroblasts [23].

The immune microenvironment is composed of T and B lymphocytes, tumor-associated macrophages (TAM), myeloid suppressor cells (MDSC), etc. These cells mediate the immune suppression microenvironment and evade immunity.

T cells are critical immune players in the TME. There are highly enriched regulatory T cells (Tregs) in the TME, which participate in tumor progression. They can directly kill effector T cells by producing cytotoxic T lymphocyte-associated antigen 4 (CTLA-4), consuming interleukin-2 (IL-2), and secreting inhibitory cytokines, thus inhibiting antitumor immunity [24]. T cell apoptosis and the p53 pathway are significantly downregulated, which may be the mechanism regulating Tregs-related immunosuppression. The upregulation of interferon (IFN)-γ and IFN-α response allows Tregs to lose their responsiveness to IFN, which is also one of the causes promoting tumor development [25].

In recent years, the regulatory role of B cells in tumor immune response has received increasing attention [26]. Regulatory B cells (Bregs), also known as B10 cells, are a subpopulation of B cells. These cells can attenuate antitumor immunity by secreting IL-10 and inhibiting the immune response of T cells, thus promoting tumor development [27]. Furthermore, IL-35 produced by B cells can inhibit the effects of effector T cells by inhibiting the response of effector CD4+ and CD8+ T cells, causing the proliferation of Treg cells [28].

TAM define the plasticity and heterogeneous cell population of the TME. Macrophages have M1 and M2 activation states, and TAMs have mainly an M2-like phenotype, showing an immunosuppressive state. Tumor cells produce a large amount of lactic acid through aerobic glycolysis, and lactic acid acts as a messenger between tumor cells and TAM to induce M2-like TAM. In turn, TAM recruit or activate endothelial cells to produce vascular endothelial growth factor A (VEGF-A), epidermal growth factor (EGF), C-X-C motif chemokine ligand 8 (CXCL8), and CXCL12 to promote tumor angiogenesis and continuously provide nutritional support for malignant cells [29, 30].

MDSC are myeloid cell populations produced under pathological conditions. These cells represent a pathological state of activation of monocytes and relatively immature neutrophils [31]. Tumor-derived exosomes (TEXs) released by the TME accelerate the activation, expansion, and immunosuppression of MDSCs. Meanwhile, expanded and activated MDSCs enhance the proliferation, angiogenesis, migration, and immune escape of cancer. MDSCs infiltrated in TME lead to resistance to cancer immunotherapy and lead to a poor prognosis for chemotherapy [32].

The non-immune microenvironment is mainly composed of stromal cells, including cancer-associated fibroblasts (CAFs), extracellular matrix (ECM), and other secretory molecules, such as growth factors, cytokines, chemokines, and extracellular vesicles, which play multiple roles in tumor occurrence and prognosis.

CAFs have multiple functions in the TME, including matrix deposition and remodeling, interaction with cancer cell signals, and crosstalk with infiltrating leukocytes [33]. STRING analysis of the CAF proteome revealed an interaction center related to collagen synthesis, modification, and signaling [34]. Dysregulation of the interaction center will form a strong tumor promoting microenvironment, which, in combination with the activation of chemokines such as CXCL1 and CXCL8, will jointly drive cancer cell invasion and promote disease progression [35].

ECM is mainly composed of proteoglycans, glycoproteins, mother cell proteins, osteopontin, thrombospondins, and various structural proteins. The remodeling feature of ECM in most tumors is that changes in the content, activity, and cross-linking of these proteins trigger changes in signal transduction, resulting in cytotoxic immune cells that primarily undergo immune elimination being influenced by the signals provided by ECM itself, and unable to kill tumor cells in the reshaped ECM [36, 37].

Unlike malignant cells that directly invade normal tissues and spread to other parts of the body using the lymphatic or circulatory system, nonmalignant cells in the TME play a cancer promoting function at all stages of carcinogenesis by stimulating uncontrolled cell proliferation (Fig. 2) [38]. Nonmalignant cells in the TME have the immunophenotype and ability to influence disease progression, which may provide new targets for the treatment of cancer.

## Application of single-cell sequencing in the diagnosis and treatment of malignant head and neck tumors

### Oral squamous cell carcinoma

Oral squamous cell carcinoma (OCSCC) is a common malignant tumors in the oral cavity. The main risk factors for OCSCC are tobacco products, alcohol, betel nut, and genetic alterations [39]. Here, we will focus on the cellular heterogeneity caused by genetic changes in OCSCC.

Human papillomavirus (HPV) is considered an independent risk factor for OCSCC [40]. E6 and E7 are the main oncogenes in HPV. When they are integrated into the host chromosome, they interfere with the function of tumor suppressor proteins. E6 binds to p53 through the E3A ubiquitin protein ligase (UBE3A) and degrades p53 through a proteasome-mediated pathway. When E7 binds to the pRb protein, it causes the separation of pRb and E2F (a transcription factor), resulting in E2F driving cells to enter the S phase of the cell cycle uncontrollably. These cell cycle regulators cannot control cell division, resulting in uncontrolled cell proliferation and ultimately cancer [41, 42].

The inactivation of p53 plays an important role in the occurrence of HPV positive OCSCC, making it widely studied as a diagnostic gene for HPV positive OCSCC. The most common mutation in OCSCC is the TP53 gene [43]. The deregulation of p53, a tumor suppressor encoded by TP53, is a very important event in the progression of OCSCC, which leads to cell cycle hyperactivation and causes cancer events [44]. Mutant p53 is not only a long-lived inactive protein, but it can also adversely affect the remaining wild-type p53 (WT-p53), the so-called dominant negative effect, to actively drive tumorigenesis as an oncogenic protein [45]. In addition, several studies have found that p53 is usually inactivated after the expression of HPV oncoprotein E6, which suggests that there may be an interaction between p53 and HPV infection [46]. In addition to TP53, overexpression of the MYC gene is also common in OCSCC patients [47]. MYC is a transcription factor that drives cell growth and proliferation, regulates more than 15% of the human genome, and controls transcription mediated by three RNA polymerases [48, 49]. However, in most cases, MYC does not act alone, but cooperates with other oncogenes, especially p53 mutations [50].

In HPV negative OCSCC, Quah et al. analyzed the correlation between AXL and AURK in OCSCC and tumor metastasis using single-cell resolution, which can serve as markers of invasive primary tumors. According to the trajectory comparison results of CD8^+^T lymphocyte population, SOX4 was identified as a driving factor for CD8 cell dysfunction and a potential marker for regulating T cell depletion [51]. Zhang et al. also found 8 gene markers associated with CD8 T cell infiltration (DEFB1, AICDA, TYK2, CCR7, SCARB1, ULBP2, STC2, and LGR5) by integrating single cell and batch RNA sequencing in head and neck squamous cell carcinoma, which can accurately predict the prognosis of OCSCC and serve as clinical treatment indicators [52]. By applying single-cell genomics and analyzing trajectories and interaction groups, we can better reveal the mechanisms of tumor occurrence and development, and search for reliable biomarkers for tumor diagnosis and prediction of clinical prognosis.

Regarding the treatment of OCSCC, immune checkpoint-mediated immunotherapy has shown great potential. Cytotoxic T lymphocyte-associated antigen 4 (CTLA-4) and programmed death 1 (PD-1)/programmed cell death ligand 1 (PD-L1) are the two most representative immune checkpoint pathways [53].

PD-1 is a co-inhibitory receptor on the surface of cytotoxic T lymphocytes. The expression of PD-1 and PD-L1 on tumor cells or antigen-presenting cells (APCs) triggers an immunosuppressive response, achieves metabolic reprogramming of T cells, reduces effector T cells and memory T cells, and increases the number of Tregs and exhausted T cells [54]. Single-cell sequencing analysis identified the CD8^+^TIL subgroup, highlighting the heterogeneity and differentiation of CD8^+^TIL, further improving the therapeutic effect of immune checkpoint blockade [55]. The use of clinically commonly used anti PD-1 monoclonal antibodies such as Navulizumab and Pembrolizumab can effectively improve the therapeutic effect and prolong the survival period of tumor patients by blocking the PD-1/PD-L1 axis. This approach has been applied to the preoperative treatment of recurrent/metastatic OCSCC [56, 57].

CTLA-4 and CD28 are homologous receptors expressed by CD4+ and CD8+ T cells. CTLA-4 has the opposite function to CD28. It competes with the CD28 costimulatory molecule for costimulatory ligands CD80 and CD86, mediates the inhibitory signal that inhibits T cell activation, weakens the early activation of naive and memory T cells, and directly reduces the ability of APC to stimulate through CD28, so that the CD28/CTLA-4 system can play a role as a rheostat and regulate T cell activation [58, 59]. Inhibition of CTLA-4 function can lead to the depletion of Treg cells in the TME, which confirms that CTLA-4 inhibitors such as ipilimumab are an effective strategy for the treatment of solid tumors [60].

With the gradual maturity of SCS technology, researchers are continuously developing new cancer adjuvant therapies in the field of cancer using this technology. For example, oncolytic viral therapy [61], vaccine treatment [62, 63], and adoptive cell therapy [64]. However, the above methods cannot work alone and need to be combined with other treatment methods.

### Nasopharyngeal carcinoma (NPC)

Nasopharyngeal carcinoma (NPC) is a disease with strong etiological association with Epstein Barr virus (EBV) [65]. Latent genes expressed by EBV genome include: Six EBV encoded nuclear antigens, latent membrane proteins LMP-1 and LMP-2A/2B, EBV encoded small RNAs (EBER1 and EBER2) and microRNAs (BHRF1 and miR-BART) [66]. The analysis of NPC samples showed that NPC was closely related to the expression of LMP-1, LMP -2, and miR-BART [67, 68]. We will focus on the mechanism of action of the aforementioned genes in the occurrence and development of tumors.

LMP-1 is a major oncogene that can change the expression profile of APOBEC3, convert cytosine to uracil in host and viral genes, cause DNA mutations, and accumulate in patients with NPC [69]. LMP-1 induced host dependent factors, which are essential for escaping EBV-induced tumor suppressive responses [70]. LMP-1 can enhance the transcription of glucose transporter 1 (GLUT1) to promote extramitochondrial glycolysis in malignant cells, and expand tumor-associated MDSCs, leading to tumor immunosuppression in NPC [71].

LMP-2A supports the function of LMP-1 to a certain extent and promotes malignant transformation of host cells through multi-point intervention in signaling pathways, especially in apoptosis and cell cycle pathways. LMP-2A can block the tyrosine phosphorylation induced by the B cell receptor, synergize with LMP-2B, and prevent the transition from latent to lytic EBV replication [72].

MicroRNAs encoded by EBV are a reliable biomarker source for the diagnosis of NPC [73]. The overexpression of circulating EBV-miR-BART7 is correlated with nodal status and tumor grade [74]. The expression of EBV-miR-BART8 in NPC is significantly higher than that in normal nasopharyngeal mucosa. EBV-miR-BART8 promotes NPC migration, invasion, and metastasis, drives the EMT process, and up-regulates the expression of metastasis-related proteins in NPC cells [75].

The imbalance of the human body’s own signaling pathways and genetic variations accelerate the process of EBV infection, thereby further promoting the progression of NPC.

Dysregulated NF-κB signaling may help establish potential EBV infection in NPC. Genomic studies of NPC using whole exome sequencing have identified multiple somatic mutations in upstream negative regulators of the NF-κB signaling pathway [76]. Besides the NF-κB signaling pathway, PI3K/Akt/mTOR signaling pathway can also promote the carcinogenic properties of EBV-infected epithelial cells [77]. Genetic variation of human leukocyte antigen (HLA) not only allows tumor cells to escape immune surveillance by immune cells [78], but also plays a crucial role in the reactivation of EBV. After primary EBV infection, the virus remains dormant for most of the time, and the HLA-DRB1 * 09 allele reactivates EBV by binding to the EBV specific glycoprotein gp42, helping EBV infect cells [79]. Moreover, the amino acid Phe-1 located in HLA-DRB 67 peptide binding pocket and Glu-45 located in HLA-B pocket B have a protective effect on NPC susceptibility. The allele rs2894207 located between HLA-B and HLA-C shows a protective effect on the occurrence of NPC [80].

Radiotherapy and chemotherapy are the main treatment options for nasopharyngeal carcinoma. However, their efficacy in patients with locally advanced or distant metastatic tumors is limited. Tumor immunotherapy is a promising treatment method for nasopharyngeal carcinoma, including vaccination, adoptive cell therapy, and immune checkpoint blockade [81].

Several preventive vaccines have been developed to prevent EBV infection. The target cells for EBV are B lymphocytes and epithelial cells. Flow cytometry, single-cell sorting, and other technologies have confirmed that the EBV envelope proteins, including gH/gL, gB, and gp350 play a key role in EBV entry and target cell infection. At the same time, these envelope proteins can also be used as candidates for EBV preventive vaccine [82]. gp350 is the most abundant glycoprotein on the surface of virus-infected cells and virus particles, and is highly conserved, so it has become the focus of vaccine development. Another vaccine induces T cells to respond to the EBV lytic protein and EBV latent protein, which can significantly reduce the incidence of disease [83, 84].

Adoptive cell transfer therapy involves enhancing the cytotoxic function of biologically active immune effector cells from patients in vitro or inducing them to differentiate into immune effector cells with antitumor activity. These cells are then injected into the patient’s body to exert their antitumor effects, including tumor infiltrating lymphocytes (TIL) therapy, chimeric antigen receptor (CAR) T cell therapy, and engineered T cell receptor (TCR) therapy, NK cell therapy [85]. Bonifacius et al. have shown that functional EBV specific cytotoxic T lymphocytes derived from autologous or stem cell donors (SCD derived) or partially HLA matched third-party donors (TPD derived) generate the EBV-CTL line through repeated in vitro stimulation of antigen carrying cells, which is well tolerated and achieves good therapeutic effects in most patients [86].

Immunocheckpoint inhibitors can disrupt immune defenses and restore endogenous antitumor immunity. EBV-induced NPCs usually exhibit overexpression of PD-1. Treprizumab is an IgG4 monoclonal antibody targeting PD-1, which has good antitumor activity in most patients. Whole exome sequencing revealed patients with genomic amplification of the 11q13 region or ETV6 genomic changes showed poor response to treparizumab [87, 88]. Pabolizumab, carrelizumab, and nabulizumab also showed good objective remission rates in the clinic [89].

Traditional Chinese medicine treatment has been proven to have certain therapeutic effects on NPC through experiments. Emodin, a common TCM, has potential anti-EBV activity. We showed previously that emodin inhibits the expression of EBV lytic protein and blocks the production of virions in EBV positive epithelial cell lines [90]. Ganlu drink for nourishing yin and clearing heat can reduce cell viability, inhibit tumor proliferation, and induce apoptosis through poly-ADP ribose polymerase and caspase-3-dependent pathway [91].

### Laryngeal squamous cell carcinoma

Laryngeal squamous cell carcinoma (LSCC) is the main histological type of laryngeal carcinoma, accounting for more than 95% of laryngeal cancer [92]. The abnormal expression of KRT16, HMGA2, metalloproteinase (MMP), and the tissue inhibitor of metalloproteinase (TIMP) was detected by laser capture microdissection and gene chip technology in laryngeal carcinoma, and may be closely related to the appearance of LSCC [93, 94].

The KRT16 gene encodes a keratinocyte hyperproliferation gene associated with cytokeratin, which can respond to stress in the endoplasmic reticulum and inhibition of the mitochondrial respiratory chain [95]. HMGA2 is expressed in almost all human malignancies, promoting cell proliferation by promoting cell cycle entry and inhibiting apoptosis [96]. The role of the extracellular matrix (ECM) in tumor growth and metastasis cannot be ignored either. MMP is considered to be one of the major enzymes involved in ECM degradation [97]. MMP can lead to the degradation of ECM proteins (such as collagen and elastin), and affects cell proliferation, metastasis, and differentiation, ECM remodeling, as well as tissue invasion and angiogenesis, contributing to the invasion of cancer cells [98]. In cancer, elevated MMP levels are associated with tumor progression and invasiveness. MMP is regulated by TIMP, and the MMP/TIMP ratio usually determines the extent of ECM protein degradation and tissue remodeling [99]. TIMP regulates angiogenesis, cell proliferation, and apoptosis, and is an important regulator of ECM turnover, tissue remodeling, and cell behavior. Therefore, disrupting the balance between MMPs and TIMPs is associated with the progression of cancer [100].

Treatment of LSCC is usually based on a combination of surgery, radiotherapy, and chemotherapy. However, some advanced cases have developed resistance to treatment and poor survival results [101]. At this time, we need to identify new treatment strategies, microRNA (miRNA) and lncRNA have become popular research directions in recent years.

MiRNAs are short non-coding RNAs that play a role in gene silencing and translation inhibition by binding to target mRNAs [102]. People use scRNA-seq technology to detect multiple miRNA expression abnormalities in LSCC [103], including but not limited to MiR-125, which reduces cell growth, migration, and invasion in LSCC cells by directly targeting signal transduction and transcription activating factor 3 (STAT3), is downregulated in expression [104]. MiR-107 has the function of inhibiting the proliferation and invasion of LSCC cells in vitro by targeting CACNA2D1, and its expression is downregulated [105]. Upregulation of miR-196b expression is associated with major clinical features and poor prognosis [106]. lncRNA are key players in LSCC progression. Abnormal expression of lncRNA was also found in LSCC [107]. LncRNA miR143HG inhibits miR-21 by methylation and inhibits invasion and migration [108]. lncRNA GAS5 inhibits LCSS cell proliferation and promotes apoptosis by targeting miR-26a-5p and modifying ULK2 [109]. These differences in miRNA and lncRNA expression levels are one of the molecular mechanisms underlying the occurrence and progression of LSCC and are considered to have great potential as biomarkers and therapeutic targets.

LSCC is a type of HNSCC, and its therapeutic drugs are similar to HNSCC. In addition to conventional cytotoxic anticancer agents, the tyrosine kinase inhibitors of the epidermal growth factor receptor (cetuximab) and immune checkpoint inhibitors such as the PD-1 antibody (nabulizumab and pabilizumab) are also used in the clinical treatment of LSCC [110]. The TCM Zao-Jiao-Ci (ZJC) is also considered a candidate drug for the treatment of LSCC. ZJC is related to the dysregulation of transcriptional signaling pathways in the tumor and cell cycle [111].

### Thyroid cancer

Thyroid cancer (THCA) is divided into several histological subtypes: papillary thyroid carcinoma (PTC), follicular thyroid carcinoma (FTC), medullary thyroid carcinoma (MTC), and anaplastic (undifferentiated) thyroid carcinoma (ATC). Using SCS technology to reveal the tumor heterogeneity of thyroid cancer provides help for the diagnosis and treatment of advanced thyroid cancer.

PTC is the most common well differentiated histological type in THCA, accounting for approximately 90% of all THCA [112]. PTCs are inert and one of the cancers with the lowest mutation density. BRAF^V600^ is the most common and specific genetic alteration in PTC [113, 114]. FTC accounts for about 5% of thyroid cancer. In FTC, the most common mutation involves the RAS gene family. In addition, the fusion gene PAX8-PPARγ was identified in one third of FTC cases [115].

Differentiated thyroid cancer (DTC) (including PTC and FTC), with surgery and radioactive iodine (RAI) treatment as the first-line treatment, usually has a good prognosis and high survival rate. However, a small number of radioactive iodine refractory (RAI-R) cases have extremely high mortality due to lack of effective treatment [116]. Anti angiogenic tyrosine kinase inhibitors are the main targets of advanced thyroid cancer [117]. Two multi kinase inhibitors (MKI), lenvatinib and sorafenib, have been applied in the treatment of advanced DTC and are considered as standard first-line treatment for radioiodine refractory differentiated thyroid cancer (RR-DTC) [118]. Lenvatinib targets VEGFR 1-3, RET, fibroblast growth factor receptor (FGFR) 1-4, platelet-derived growth factor receptor (PDGF)a. Sorafenib targets VEGFR 1-3, RET, BRAF, and PDGFb [119]. In addition, cabotinib can also be used as a remedial treatment for tyrosine kinase inhibitor RR-DTC [120].

Approximately 2–3% of thyroid cancer is derived from calcitonin producing C cells, known as MTC [121]. RET and RAS mutations are the main pathogenic features of MTC [122]. RET proto-oncogene is the most common driver gene in MTC [123], and the RET Met918Thr mutation is the most common [124], and is mainly caused by single amino acid substitution, insertion and/or deletion, which is a typical feature of hereditary and sporadic medullary thyroid carcinoma [125]. Ras mutations are common in RET negative tumors [126]. Ras and RET appear to be mutually exclusive, but together play a key pathogenic role in MTC. Vandetanib and cabotinib have good clinical performance in the targeted treatment of RAI refractory MTC [127]. Both drugs inhibit RET kinase activity, through VEGFR2, it has a strong inhibitory effect on key angiogenic pathways. Exploratory analysis showed that patients with RET M918T positive tumors may obtain better therapeutic effects with cabotinib [128]. For the cases of nevenditanib and cabozantinib, MKT-077 (a water-soluble anthocyanin dye analog) can effectively inhibit MTC cells by inhibiting mitochondrial molecular chaperone HSPA9 to disrupt mitochondrial bioenergetics and subsequently inducing cell apoptosis and RET downregulation, which has therapeutic potential for MTC [129]. Moreover, the selective RET inhibitors cerpatinib and pratinib are also undergoing phase I/II clinical trials, respectively, and are expected to become new antitumor drugs [130].

ATC is a rare malignant tumor that accounts for 1–2% of all thyroid cancers. It is one of the most aggressive cancers with a high mortality rate [131]. DNA mismatch repair (MMR) defects and increased activity of the APOBEC cytidine deaminase family are two major mechanisms by which ATC acquires a high mutation burden [132]. Reduced mRNA levels of cyclin dependent kinase inhibitor 2A (CDKN2A)/2B were found in ATC with repeated overexpression of APOBEC, as well as high-frequency mutation events in BRAF, TP53, TERT promoter, RAS, and other genes [133, 134]. In recent years, treatment strategies for BRAF^V600E^ have been continuously developed. BRAF^V600E^ reduces mTOR activity by regulating the activity of the AMPK pathway, thereby inhibiting the protective autophagy of thyroid cancer cells [135]. The combination of the BRAF inhibitor darafenib and the MEK inhibitor trametinib was the first therapy that showed strong clinical activity and good tolerability in the treatment of ATC with BRAF^V600E^ mutation [136]. However, it still does not meet the treatment needs of wild-type BRAF patients. Spartanzumab (PDR001) is an artificial immunoglobulin 4 monoclonal antibody that blocks the interaction with PD-L1 and PD-L2 by binding to PD-1. PDR001 is well tolerated in the ATC population and provides a feasible treatment for pd-L1-positive advanced ATC patients, including BRAF wild-type population [137]. In addition, the combination treatment of lenvatinib and pemzumab also showed good efficacy in clinical trials involving patients with ATC [138].

## Conclusions

In recent years, SCS technology has received extensive attention. With its ability to deconstruct cell subpopulations, it has become a leading technology in the investigation of head and neck malignancies. SCS technology has shown great potential in revealing the cellular heterogeneity of solid tumors and the tumor microenvironment, studying the evolution of tumor cells, invasion, diagnosis, and treatment (Table 1).

**Table 1:** Application of single-cell sequencing in malignant head and neck tumors.

Disease		Pathogenic factors	Oncogenic genes/proteins/pathways	(Potential) Therapeutic targets	Therapeutic drugs	References
OCSCC		HPV	E6, E7, MYC, p53	PD-1/L1, CTLA-4	Nabulizumab, pembrolizumab, ipilimumab	[39–64]
NPC		EBV	LMP-1, LMP-2, miR-BART, HLA	gp350, PD-1	Treprizumab, pabolizumab, carrilizumab, nabulizumab, emodin, ganluyin	[65–91]
LSCC		HPV	KRT16, HMGA2, MMP, TIMP	miR-107/125b/196b, lncRNA miR143HG, lncRNA GAS5, EGF, PD-1	Cetuximab, nabulizumab, pabolizumab, ZJC	[92–111]
THCA	PTC	/	BRAF^V600^	VEGFR 1-3, RET, BRAF, (FGFR) 1-4, KIT, PDGFa/b	Lenvatinib, sorafenib, cabotinib	[112–115]
	FTC	/	RAS, PAX8-PPARγ	VEGFR 1-3, RET, BRAF, (FGFR) 1-4, KIT, PDGFa/b	Lenvatinib, sorafenib, cabotinib	[116–120]
	MYC	/	RET, RAS	RET, VEGFR2	Vandetanib, cabotinib, selpatinib, pratinib, MKT-077	[121–130]
	ATC	/	CDKN2A/2B, BRAF, RAS, TP53, TERT promoter	BRAF, MEK, PD-1	Darafinib, trametinib, PDR001, pemzumab	[131–138]

SCS technology has shown great potential in revealing the cellular heterogeneity of solid tumors and the tumor microenvironment, studying the evolution of tumor cells, invasion, diagnosis, and treatment. It applies to oral squamous cell carcinoma (OCSCC), nasopharyngeal carcinoma (NPC), laryngeal squamous cell carcinoma (LSCC), and thyroid cancer (THCA).

Although SCS technology still has some problems, it can be predicted that with the reduction of costs and the development of large-scale and high throughput, this technology will play an increasingly important role, and will providing a new approach to fully understand the pathogenesis of head and neck malignancies, to carry out disease diagnosis and treatment, and to define prognosis, and thus, will actively translate scientific research to clinical application.
